# Sharp Transformation across Morphotropic Phase Boundary in Sub‐6 nm Wake‐Up‐Free Ferroelectric Films by Atomic Layer Technology

**DOI:** 10.1002/advs.202302770

**Published:** 2023-09-27

**Authors:** Chun‐Ho Chuang, Ting‐Yun Wang, Chun‐Yi Chou, Sheng‐Han Yi, Yu‐Sen Jiang, Jing‐Jong Shyue, Miin‐Jang Chen

**Affiliations:** ^1^ Department of Materials Science and Engineering National Taiwan University Taipei 10617 Taiwan; ^2^ Research Center for Applied Sciences Academia Sinica Taipei 11529 Taiwan

**Keywords:** morphotropic phase boundary, antiferroelectricity/ferroelectricity, atomic layer technology, phase transition, helium ion microscopy

## Abstract

Atomic layer engineering is investigated to tailor the morphotropic phase boundary (MPB) between antiferroelectric, ferroelectric, and paraelectric phases. By increasing the HfO_2_ seeding layer with only 2 monolayers, the overlying ZrO_2_ layer experiences the dramatic phase transition across the MPB. Conspicuous ferroelectric properties including record‐high remanent polarization (2*P_r_* ≈ 60 µC cm^−2^), wake‐up‐free operation, and high compatibility with advanced semiconductor technology nodes, are achieved in the sub‐6 nm thin film. The prominent antiferroelectric to ferroelectric phase transformation is ascribed to the in‐plane tensile stress introduced into ZrO_2_ by the HfO_2_ seeding layer. Based on the high‐resolution and high‐contrast images of surface grains extracted precisely by helium ion microscopy, the evolution of the MPB between tetragonal, orthorhombic, and monoclinic phases with grain size is demonstrated for the first time. The result indicates that a decrease in the average grain size drives the crystallization from the tetragonal to polar orthorhombic phases.

## Graphical abstract

Significant ferroelectricity in sub‐6 nm thin films is boosted by atomic layer engineering on the HfO_2_ seeding layer, triggering the morphotropic phase boundary effect correlated with the dramatic transition from antiferroelectric to ferroelectric phase in ZrO_2_. Furthermore, clear images of surface grains taken by helium ion microscopy are employed to characterize the transformation of crystallographic structures for the first time.

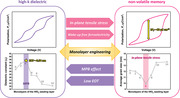

## 1 Introduction

The morphotropic phase boundary (MPB) between the antiferroelectric (AFE) and ferroelectric (FE) phases is a decisive factor to tailor the crystalline structures, which has caught widespread spotlight and interest recently.^[^ ^1^ ^]^ The discovery of AFE and FE properties in HfO_2_/ZrO_2_‐based thin films, which are derived from the non‐polar tetragonal phase (t‐phase) and polar orthorhombic phase (o‐phase),^[^ ^2^ ^]^ respectively, has boosted rapid development of MPB in extensive research fields and practical applications.^[^ ^3^ ^]^ In comparison with conventional perovskite materials, the fluorite HfO_2_/ZrO_2_‐based materials have favorable advantages including higher remanent polarization even with a thickness down to sub‐10 nm, larger bandgap, environmental friendliness, and high compatibility with advanced semiconductor technology.^[^ ^4^ ^]^ The distinguished superiority makes the HfO_2_/ZrO_2_‐based thin films very attractive in many fields such as high‐k gate dielectrics,^[^ ^5^ ^]^ non‐volatile memory devices,^[^ ^2c^ ^]^ energy‐storage devices,^[^ ^6^ ^]^ and so on.

With the evolution of 3D stacked memory structures, the continuous thickness scaling of FE thin films is urgently demanded to further increase the memory density.^[^ ^7^ ^]^ In addition, concerning the integration with the back‐end‐of‐line (BEOL), the processing temperatures for fabricating the FE devices should be restricted below 400 °C to reduce the impact of high thermal budget.^[^ ^8^ ^]^ However, in terms of various dopants such as Zr, Si, Al, Y, and La in well‐known high‐performance HfO_2_‐based FE thin films,^[^ ^9^ ^]^ the post‐annealing temperatures generally exceed 550 °C, even up to 800 °C, as the film thickness is lower than 6 nm,^[^ ^10^ ^]^ which is incompatible with the BEOL processes. The thickness scaling of the HfO_2_‐based thin films to the sub‐6 nm regime also gives rise to significant degradation of the ferroelectricity, which is ascribed to the increasing proportion of the dead layer (non‐polar phases) in the film.^[^ ^11^ ^]^ As a consequence, the improvement of crystallization into the polar o‐phase is regarded as a challenge to the FE thin films with a thickness scaling below 6 nm.

One of the promising strategies to boost the ferroelectricity in sub‐6 nm thin films is the MPB‐correlated AFE to FE phase transition. It has been well recognized that the t‐phase is the most stable crystallographic structure in nanoscale ZrO_2_ thin films.^[^ ^2^, ^12^ ^]^ The relatively low crystallization temperature gives ZrO_2_ an edge over the HfO_2_‐based materials because of its more compatibility with BEOL.^[^ ^13^ ^]^ Therefore, it is of great significance to explore the pathway to induce the phase transformation from the non‐polar t‐phase to the polar o‐phase in ZrO_2_ thin films. It has been pointed out that the presence of MPB causes the transition between the AFE and FE phases by adjusting the composition, crystallization temperature, applied electric field, etc.^[^ ^1^, ^3^ ^]^ In this research, a novel method is proposed for achieving the AFE to FE phase transformation by manipulating the MPB through monolayer engineering on a nucleation seeding layer. The various types of seeding layers such as Al_2_O_3_, TiO_2_, ZrO_2_, and HfO_2_ have been intensively investigated to improve the FE performance of nanoscale Hf_0.5_Zr_0.5_O_2_ (HZO) thin films.^[^ ^14^ ^]^ The seeding layer can facilitate the crystallization of the polar o‐phase, reduce the leakage current, adjust the dielectric properties, and improve the cycling reliability of HZO thin films.^[^ ^14^ ^]^ Although the studies have been conducted so far, there is still much room for significant improvement in ferroelectricity. Critical issues need to be addressed, including the thickness scaling, the reduction of process temperature, and the need for the wake‐up operation. As opposed to the comprehensive research on HZO thin films, the literature about the impact of the seeding layer on nanoscale ZrO_2_ thin films is scarce. In order to achieve pronounced FE properties in sub‐6 nm thin films, the atomic layer engineering on the HfO_2_ nucleation seeding layer is used to tailor the MPB for the AFE to FE phase transition in the overlying ZrO_2_ layer in this paper.

On the other hand, the prerequisite wake‐up process to achieve the optimal ferroelectricity in HZO devices is considered a practical challenge and a deterioration in the device reliability.^[^ ^15^ ^]^ The wake‐up effect has been recognized to be relevant with the conversion from the non‐polar crystalline phases to the polar o‐phase or the depinning of FE domains in the HZO thin films.^[^ ^16^ ^]^ The charged defects such as oxygen vacancies are redistributed more uniformly after the implementation of the cycling bipolar electric field (i.e., the wake‐up process), which leads to a homogeneous internal electric field for the reduction in the energy barrier of the transition from the non‐polar to the o‐phases.^[^ ^15^, ^16^ ^]^ The formation of oxygen vacancies may be suppressed by the enhancement of crystal quality since high crystallinity can effectively confine the flexibility of lattice structures and restrain atomic relaxation.^[^ ^17^ ^]^ The monolayer engineering on the HfO_2_ seeding layer in this work might increase the degree of crystallinity, which is highly favorable for a decrease in the content of oxygen vacancies in the ZrO_2_ layer to alleviate the wake‐up effect, leading to the wake‐up‐free operation in various FE devices including ferroelectric field‐effect transistors,^[^ ^18^ ^]^ ferroelectric random access memories,^[^ ^19^ ^]^ and ferroelectric tunnel junctions.^[^ ^20^ ^]^

In this paper, the MPB phenomenon for the transformation between the AFE, FE, and paraelectric (PE) crystalline phases in nanoscale ZrO_2_ is investigated in detail by atomic layer engineering on the HfO_2_ seeding layer. The monolayer tailoring of materials is realized by the self‐limiting chemical reactions at the surface in the atomic layer deposition (ALD) process. In addition, the comprehensive electrical and material characterizations are conducted carefully to explore the MPB‐related phase transition, including the AFE/FE hysteresis loops, dielectric constant, high‐resolution transmission electron microscopy (HRTEM), nano beam diffraction (NBD), and X‐ray photoelectron spectroscopy (XPS). The out‐of‐plane, grazing incident, and in‐plane high‐power X‐ray diffraction (XRD) measurements and the sin^2^ *ψ* method in the grazing incident configuration are also utilized to probe the residual strains in ZrO_2_ induced by the HfO_2_ seeding layer, revealing the correlation of MPB with the in‐plane tensile stress. Especially, the impacts of the grain size and its distribution on the AFE, FE, and PE phase transformation are analyzed using helium ion microscopy (HIM) for the first time to capture high‐resolution and high‐quality surface images. Conventionally, scanning electron microscopy (SEM) is a widely used method to acquire plane‐view images. However, it is inappropriate for SEM to characterize non‐conductive thin films due to the accumulation of charges at the surface. Since the charging effect causes blurred surface images of non‐conductive thin films,^[^ ^12^, ^21^ ^]^ coating a heavy metal thin film such as gold or platinum is necessary to mitigate the surface charging in SEM. Nevertheless, the heavy metal coating leads to a distortion of the surface morphology or results in a loss of subtle surface features.^[^ ^21a^ ^]^ Benefiting from the larger mass of He ions in HIM than that of electrons used in SEM, the scattered He ions keep almost the same direction when penetrating into the sample.^[^ ^22^ ^]^ Therefore, the interaction area on the sample surface is more convergent in HIM than that in SEM, giving rise to a much smaller spot size, higher resolution, and greater depth of focus in HIM.^[^ ^22^, ^23^ ^]^ Besides, the issue of the charging effect could be avoided because of the deeper penetration depth of He ions and the neutralization by the electron flood gun equipped in HIM.^[^ ^21^, ^22^ ^]^ As a consequence, the surface charging effect can be considerably alleviated in non‐conductive samples without any heavy metal coating in HIM, and so real images of tiny surface structures can be acquired. Moreover, a higher yield of secondary electrons further enhances the contrast of HIM images, which is conducive to clearly defining grain sizes and boundaries on the sample surface.^[^ ^24^ ^]^

Based on the precise atomic layer engineering on the HfO_2_ seeding layer in this study, the dramatic AFE to FE phase transition in ZrO_2_ is achieved at a low processing temperature of only 400 °C, which leads to the realization of robust ferroelectricity with a record‐high remanent polarization (2*P_r_* ≈ 60 µC cm^−2^) in sub‐6 nm FE thin films, along with being free of the wake‐up operation. The excellent FE performance and the low thermal budget render the HZ (ZrO_2_ on the HfO_2_ seeding layer) structure indispensable and highly anticipated in nonvolatile memory devices in advanced semiconductor technology nodes.

## 2 Results and Discussion

### 2.1 The Sample Structure for Monolayer Engineering

**Figure 1:**
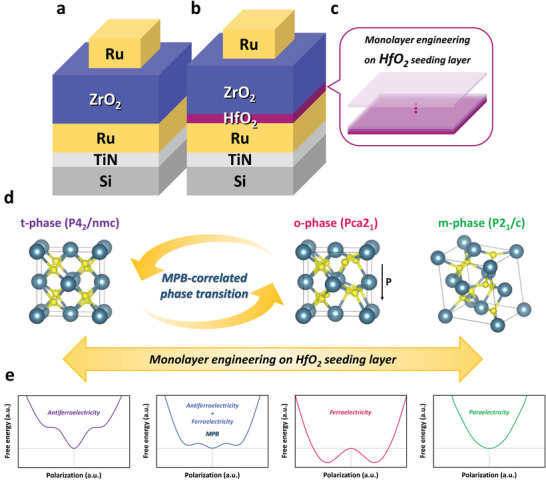
The schematic diagrams of the MIM devices and the phase transition in the nanoscale ZrO_2_ layer. a) The H_0_Z device, and b) the H_2_Z, H_4_Z, H_6_Z, H_8_Z, H_10_Z, and H_14_Z devices in which the ALD cycles for depositing the HfO_2_ seeding layer range from 2 to 14. c) The thickness control of atomic‐level resolution, which is terminologically labeled as “monolayer engineering,” is conducted on the HfO_2_ seeding layer. d) Transformation of the ZrO_2_ crystalline structures modulated by the HfO_2_ seeding layer, and especially, the transition between the t‐ and o‐phases is based on the presence of the MPB. e) The Landau free energy landscapes of AFE, MPB, FE, and PE characteristics.

AFE, FE, and dielectric properties of the HZ structure were examined by metal‐insulator‐metal (MIM) devices using ruthenium (Ru) as the electrodes, which are schematically plotted in **Figure** **1**a,b. 30 ALD cycles were used to deposit the ZrO_2_ layer, and the monolayer engineering on the HfO_2_ seeding layer was realized by the accurate thickness control of atomic resolution to modulate the crystalline phases in the ZrO_2_ layer as the schematic illustration in Figure 1c. The HfO_2_ seeding layer was prepared with 0, 2, 4, 6, 8, 10, and 14 ALD cycles in this paper, and so the stacking structures are denoted as H_0_Z, H_2_Z, H_4_Z, H_6_Z, H_8_Z, H_10_Z, and H_14_Z, respectively. The impact of the monolayer engineering by ALD to induce the AFE, FE, and PE phase transformation is illustrated in terms of crystal structures coupled with the Landau‐free energy landscapes in Figure 1d,e.

### 2.2 AFE and FE Characteristics

**Figure 2:**
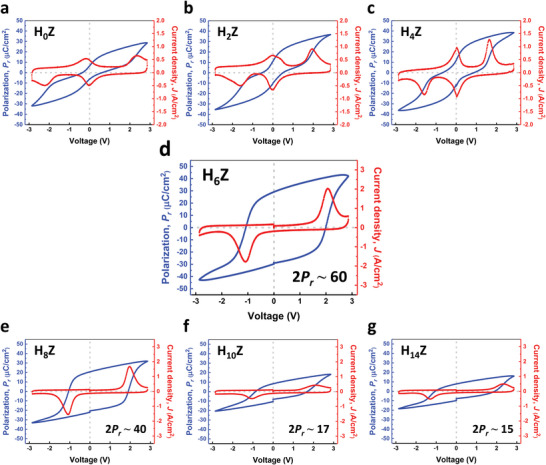
The pristine‐state *P–V* and *J–V* curves of all the MIM devices. (a–c) show the AFE characteristics in the H_0_Z, H_2_Z, and H_4_Z devices, and (d–g) present ferroelectricity in the H_6_Z, H_8_Z, H_10_Z, and H_14_Z samples, respectively. The sharp AFE to FE phase transition occurs when the *T*_HfO2_ increases from 4 to 6 ALD cycles, as shown in (c) and (d). The giant 2*P_r_* ≈ 60 µC cm^−2^ is seen in the H_6_Z sample.

The polarization versus voltage (*P–V*) and current density versus voltage (*J–V*) characteristics of all the MIM devices are shown in **Figure** **2**. Notice that all the samples were measured at the pristine state without experiencing any wake‐up process. As shown in Figure 2a–c, the pinched hysteresis loops and the quadruple peaks of switching current in the *P–V* and *J–V* curves reveal the AFE feature of the H_0_Z, H_2_Z, and H_4_Z samples. The observed remanent polarization in the pinched *P–V* curves suggests the coexistence of the AFE and FE crystalline phases, that is, the presence of MPB, in the thin films.^[^ ^25^ ^]^ It is noticeable that the remanent polarization increases from the H_0_Z to H_4_Z samples, implicating that an increase in the thickness of the HfO_2_ seeding layer (*T*_HfO2_) results in an increase of the FE phase in the HZ structures.

The sharp change in the electrical properties of the HZ thin films can be observed in Figure 2c,d as the ALD cycles of the HfO_2_ seeding layer increase from 4 to 6. Comparing the H_4_Z and H_6_Z samples manifests that the *P–V* and *J–V* characteristics were dramatically converted from the AFE to FE behaviors, which indicates that the MPB‐correlated AFE to FE phase transition occurs with an increase of *T*_HfO2_ by only 2 monolayers of HfO_2_. With a further increase in *T*_HfO2_ from 8 to 14 ALD cycles, the H_8_Z, H_10_Z, and H_14_Z devices also present the FE characteristics with typical hysteresis loops in the *P–V* curves and double switching peaks in the *J–V* curves as shown in Figure 2e–g. However, significant degradation of ferroelectricity can be observed in the H_8_Z, H_10_Z, and H_14_Z samples as the *T*_HfO2_ exceeds 6 ALD cycles. (Section S1, Supporting Information shows the FE characteristics of the H_8_Z, H_10_Z, and H_14_Z devices with higher applied voltages to ensure full switching of polarization.) Thus it can be concluded that the optimal and robust ferroelectricity is achieved in the H_6_Z sample, of which the double remanent polarization (2*P_r_*) is as high as ≈60 µC cm^−2^. It is worth noting that the 2*P_r_* ≈ 60 µC cm^−2^ is the record high in nanoscale FE thin films of a thickness below 6 nm. The result demonstrates the significant impact on the AFE and FE phase transformation by the monolayer layer engineering on the seeding layer. The polarity asymmetry of the *P–V* curves is discussed in Section S2, Supporting Information.

### 2.3 Cross‐Sectional Structures and Identification of Crystalline Phases

**Figure 3:**
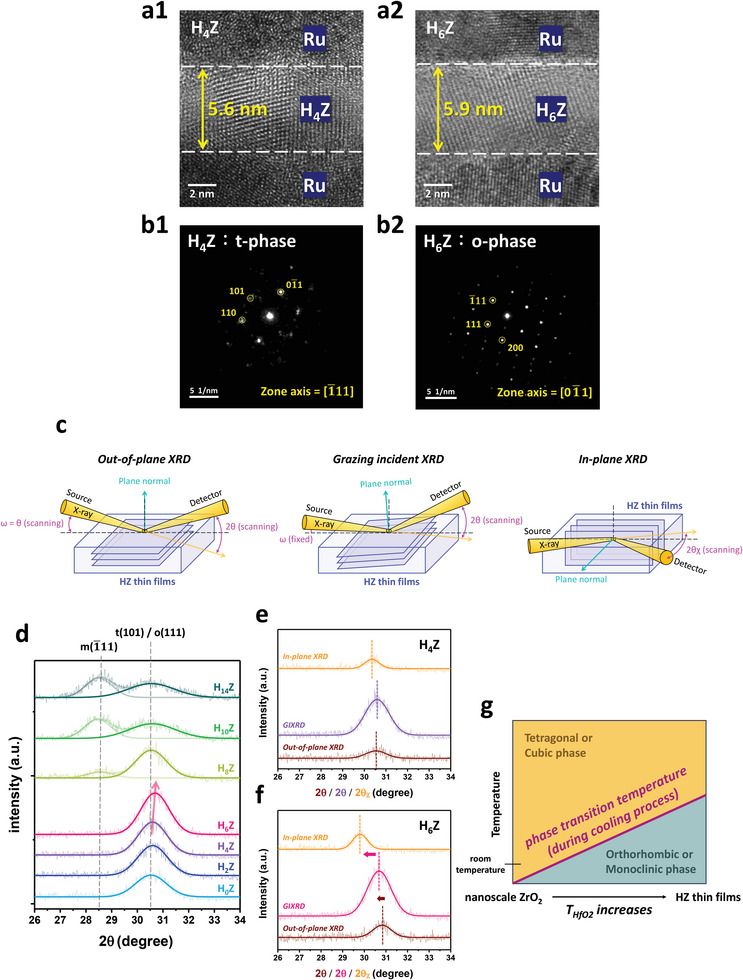
Cross‐sectional HRTEM images, NBD patterns, and crystalline structures characterized by XRD in different scanning modes. The cross‐sectional HRTEM images of the a1) H_4_Z and a2) H_6_Z samples reveal that the thicknesses of the H_4_Z and H_6_Z layers are ≈5.6 and ≈5.9 nm, respectively. b1) shows the NBD pattern of the H_4_Z layer, which is in good agreement with that of the t‐phase. b2) displays the NBD pattern of the H_6_Z layers, which is well consistent with that of the o‐phase. c) The configurations of the out‐of‐plane, grazing incident, and in‐plane XRD measurements. d) The grazing incident XRD patterns and the fitting curves of all the samples. The emergence of the t(101)/o(111) peaks suggests the coexistence of the t‐ and o‐phases in the H_0_Z, H_2_Z, and H_4_Z samples. An obvious shift in the t(101)/o(111) diffraction peak indicates the existence of residual stress in the H_6_Z sample. The m($\bar{1}$11) peak appears in the H_8_Z sample and then dominates over the t(101)/o(111) peak in the H_10_Z and H_14_Z samples. The out‐of‐plane, grazing incident, and in‐plane XRD patterns of the e) H_4_Z and f) H_6_Z samples. A small shift in the XRD peaks in (e) reveals a minor residual stress in the H_4_Z sample. However, the significant displacement of the XRD peaks in (f) indicates that the in‐plane tensile stress is present in the H_6_Z sample. The out‐of‐plane, grazing incident, and in‐plane XRD patterns of all the samples are shown in Section S3, Supporting Information. g) Schematic diagram for the temperature‐dependent phase transition in nanoscale ZrO_2_ and HZ thin films during the cooling process after annealing. In the nanoscale ZrO_2_ (H_0_Z), H_2_Z, and H_4_Z thin films, the t‐phase is predominant due to the lower phase transition temperature from the t‐/c‐ to o‐ or m‐phases. As the *T*_HfO2_ increases, the increase of the phase transition temperature leads to an increase in the content of the o‐ or m‐phase in the H_6_Z, H_8_Z, H_10_Z, and H_14_Z structures.

**Figure** **3**a1,a2 displays the cross‐sectional HRTEM images of the H_4_Z and H_6_Z samples, and the clear lattice fringes reveal a high degree of crystallinity in the films. It can be seen that the thicknesses of the H_4_Z and H_6_Z layers are ≈5.6 and 5.9 nm, respectively, which are in good agreement with the estimation based on the growth rate per ALD cycle of ZrO_2_ and HfO_2_. The NBD patterns of the H_4_Z and H_6_Z thin films are shown in Figure 3b1,b2, and the information of crystalline structures can be known from the clear NBD spots. It can be seen from Figure 3b1 that the NBD spots match well with the simulated diffraction pattern (by the CaRine software) of the *P4_2_/nmc* space group along the [$\bar{1}$11] zone axis, suggesting the presence of the non‐polar t‐phase in the H_4_Z layer. Whereas, the NBD spots of the H_6_Z thin film correspond well to the *Pca2_1_* space group along the [0$\bar{1}$1] zone axis in Figure 3b2, revealing the existence of the polar o‐phase. Hence one can conclude from the electrical characterizations and the NBD analyses (Figures 2 and 3b1,b2) that an increase by only 2 monolayers of the HfO_2_ seeding layer triggers the t‐ to o‐phase transition in the overlying ZrO_2_ layer.

The XRD patterns were measured with the out‐of‐plane, grazing incident, and in‐plane scanning modes, from which specific crystallographic planes with the normals perpendicular, inclined at an angle of ≈14.8^°^, and almost parallel to the sample surface can be probed by different configurations of the incident X‐ray and detector, as illustrated in Figure 3c. Figure 3d shows the grazing incident XRD patterns of all the samples, in which the diffraction peaks were deconvoluted with the Gaussian lineshapes. For the H_0_Z sample, the XRD peak emerges at the 2*θ* ≈ 30.5^°^, which is related to the t(101)/o(111) diffraction peak of ZrO_2_.^[^ ^26^ ^]^ The increase in *T*_HfO2_ leads to a shift of the diffraction peak toward the higher 2*θ* direction for the H_2_Z, H_4_Z, and H_6_Z samples. Despite that, the XRD peaks of the t‐ and o‐phases are difficult to separate at 2*θ* ≈ 30.5^°^; however, it has been indicated that the diffraction angle of the o‐phase is smaller than that of the t‐phase.^[^ ^27^ ^]^ Therefore, the shift in the XRD peak toward the higher 2*θ* direction is unexpected for the H_6_Z sample because of the higher degree of crystallinity into the o‐phase as revealed by its outstanding ferroelectricity as shown in Figure 2d. As a result, one can deduce that the diffraction peak shift may result from the presence of residual stress by increasing *T*_HfO2_.^[^ ^28^ ^]^

To deeply investigate the offset in the diffraction peak of the H_6_Z sample, the out‐of‐plane and in‐plane XRD patterns of the H_4_Z and H_6_Z samples were detected, which are shown in Figure 3e,f along with their grazing incident XRD patterns. It can be seen from Figure 3e that the diffraction angles of the out‐of‐plane, grazing incident, and in‐plane XRD peaks are almost identical, demonstrating that the residual stress is small in the H_4_Z sample. However, there is an obvious deviation between the out‐of‐plane, grazing incident, and in‐plane diffraction peaks in the H_6_Z sample as shown in Figure 3f. The diffraction pattern obtained from the out‐of‐plane XRD mode is associated with the t(101)/o(111) plane parallel to sample surface, and the grazing incident XRD peak stems from the t(101)/o(111) plane nearly parallel (with a small angle deviation of ≈14.8^°^) to the sample surface, whereas the diffraction peak detected from the in‐plane XRD measurement is attributed to the t(101)/o(111) plane almost perpendicular to the film surface. As compared with the out‐of‐plane and grazing incident XRD peak in Figure 3f, the lower 2*θ* of the in‐plane XRD peak indicates a larger interplanar distance (*d*‐spacing) of the t(101)/o(111) plane in the in‐plane direction, thus revealing the presence of the in‐plane tensile stress in the ZrO_2_ layer of the H_6_Z sample. The result can be understood by the surface energy effect as predicted by the thermodynamical theoretical studies, which indicates that the ≈1 nm HfO_2_ thin film is prone to form the polar o‐phase.^[^ ^12^, ^29^ ^]^ Since the lattice constant of the o‐phase in HfO_2_ is larger than that of the t‐phase in ZrO_2_,^[^ ^30^ ^]^ in‐plane tensile stress is introduced into the overlying ZrO_2_ layer. The residual tensile stress in the in‐plane direction is favorable for the boost of the phase transformation from the t‐ into o‐phases as reported in the literature,^[^ ^12^, ^31^ ^]^ and accordingly the ZrO_2_ layer experiences the transition into the polar o‐phase.^[^ ^30^, ^32^ ^]^ Therefore, the XRD patterns (Figure 3f) and the *P–V*/*J–V* curves (Figure 2d) indicate that the residual in‐plane tensile stress can effectively facilitate the crystallization of the polar o‐phase, resulting in the significant ferroelectricity in the H_6_Z sample.^[^ ^28^, ^33^ ^]^

As the ALD cycles for preparing the HfO_2_ seeding layer increase beyond 6 cycles, the grazing incident XRD peaks located at 2*θ* ≈ 28.5^°^ (attributed to the ($\bar{1}$11) plane of the monoclinic (m‐) phase (space group: *P2_1_/c*)^[^ ^26^ ^]^) emerge in H_8_Z and then dominate in the H_10_Z and H_14_Z samples as shown in Figure 3d. The result is consistent with those reported in numerous studies, which have confirmed that an increase in the HfO_2_ thickness facilitates the formation of the non‐polar m‐phase.^[^ ^30^, ^34^ ^]^ Actually, Y. Wang et al. have reported the observation of the m‐phase in HfO_2_ with a thickness of 2.48 nm,^[^ ^35^ ^]^ which is extremely close to the *T*_HfO2_ (≈2.3 nm) of the H_14_Z sample. Therefore, the substantial degradation of 2*P_r_* in the H_8_Z, H_10_Z, and H_14_Z samples can be ascribed to the suppression of the crystallization to the o‐phase in the overlying ZrO_2_ layer.

To further understand the impact of the *T*_HfO2_ on the crystalline phase in the overlying ZrO_2_ layer, a model based on the phase transition temperatures of HfO_2_ and ZrO_2_ is proposed as illustrated in Figure 3g. According to the thermodynamic models of HfO_2_ and ZrO_2_, the t‐/c‐phase is initially formed by the annealing treatment, followed by a transition from the t‐/c‐ to the m‐phases during the cooling process.^[^ ^32^, ^36^ ^]^ However, it has also been reported that the metastable o‐phase may be present as the samples are cooled down.^[^ ^36^, ^37^ ^]^ The emergence of the o‐phase is attributed to a lower kinetic energy barrier between the t‐/c‐ and o‐phases than that between the t‐/c‐ and m‐phases, which can be deduced from the effects of dopants, interface strain, surface energy, film thickness, and so on.^[^ ^36^, ^37^, ^38^ ^]^ Compared to HfO_2_, the transition temperature between the t‐/c‐ and m‐phases is much lower in ZrO_2_.^[^ ^36^ ^]^ Therefore, it can be expected that an increase in *T*_HfO2_ leads to an increase in the phase transition temperature in the HZ thin film, as depicted in Figure 3g. As a result, during the cooling process, phase transformation is more prone to take place in the HZ structure than in pure ZrO_2_.^[^ ^36^ ^]^ Therefore, the content of the o‐ or m‐phase increases as the *T*_HfO2_ increases in the HZ thin film.

### 2.4 Sin^2^ *ψ* Method for the Residual Stress Analysis Based on the Grazing Incident Configuration

**Figure 4:**
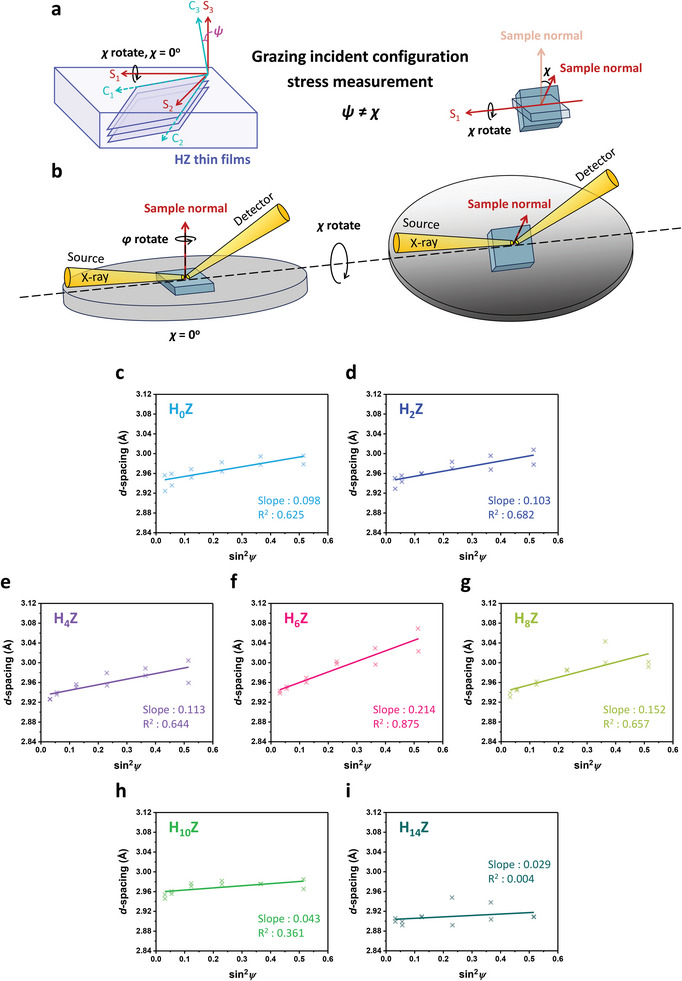
The *d*‐spacing versus sin^2^ *ψ* plots of the residual stress measurement. a) The orthogonal coordinate systems of the sample surface (S_1_, S_2_, S_3_) and the crystal plane (C_1_, C_2_, C_3_). b) The sketch of the grazing incident configuration for the stress measurement. The *d*‐spacing as a function of sin^2^ *ψ*, along with the regression line, of the c) H_0_Z, d) H_2_Z, e) H_4_Z, f) H_6_Z, g) H_8_Z, h) H_10_Z, and i) H_14_Z samples.

The more rigorous analysis of residual stress in materials involves using XRD to measure the *d*‐spacing of specific crystallographic planes with different orientations.^[^ ^39^ ^]^ As shown schematically in **Figure** **4**a,b, two orthogonal coordinate systems, that is, (S_1_, S_2_, S_3_) on the sample surface and (C_1_, C_2_, C_3_) upon the crystal plane, are employed to describe the experimental configuration. The *ψ* (psi) is the angle between the normals of the sample surface (S3) and the crystal plane (C3), and the *χ* (chi) is the angle of tilting the sample normal along the S1 axis. One of the approaches to extract the residual stress is known as the “sin^2^ *ψ* method,”^[^ ^39^, ^40^ ^]^ which employs the standard Bragg–Brentano configuration (the symmetric diffraction geometry, *ω = θ* and *ψ = χ*) with the variation in the *ψ* angle. However, the sin^2^ *ψ* method is not appropriate for stress analysis of thin films because significant penetration of the X‐ray irradiation into the substrate greatly reduces the diffraction intensity from the thin film.^[^ ^39^, ^41^ ^]^ An alternative method that is advantageous for thin films is the grazing incident configuration with the asymmetric diffraction geometry (*ω*≠*θ* and *ψ*≠*χ*) as shown in Figure 4a,b,^[^ ^41^, ^42^ ^]^ where the relationship between the *ψ* and *χ* angles is given by:^[^ ^41^ ^]^

(1) $$\cos\;\psi \;=\;\cos\left(\omega -\theta \right)\;\cos\;\chi$$

If residual stress is present in a thin film, the *d*‐spacing of specific diffraction planes will vary as measured at different *ψ* angles. Due to the effect of Poisson's ratio,^[^ ^43^ ^]^ when a thin film is subjected to an in‐plane tensile stress, a corresponding compressive stress will be generated in the out‐of‐plane direction. Figure 4c–i shows the stress measurement of the t(101)/o(111) diffraction planes for *χ* = 0^°^, 9^°^, 18^°^, 27^°^, 36^°^, and 45^°^ using the grazing incident configuration, with *φ* = 0^°^ and 180^°^ at each *χ* angle (*φ* (phi) is the azimuth angle of rotating around the normal of the sample surface as plotted in Figure 4b). As a result of the ultrathin film thickness, the t(101)/o(111) diffraction is almost undetectable as *χ* is greater than 45^°^. The *d*‐spacing at different sin^2^ *ψ* is derived from the 2*θ* position of the XRD peak. It can be seen that the slopes of the regression lines are 0.098, 0.103, and 0.113, respectively, for the H_0_Z, H_2_Z, and H_4_Z samples. The slight increase in the slope reveals a gentle rise in the in‐plane tensile stress as the number of monolayers in the HfO_2_ seeding layer increases from 0 to 4. It is noteworthy that there is a steep increase in the slope to 0.214, accompanied by a maximum coefficient of determination (*R* ^2^) of 0.875, for the H_6_Z sample. This indicates the existence of substantial and homogeneous in‐plane tensile stress, contributing to significant ferroelectricity of the H_6_Z device. Compared to the H_6_Z sample, the slope (0.152) and the *R* ^2^ value drop in the H_8_Z sample, manifesting a release of residual stress that is preferable for the emergence of the m‐phase as widely reported in the literature.^[^ ^11^, ^44^ ^]^ As the *T*_HfO2_ further increases, the m‐phase becomes dominant in the H_10_Z and H_14_Z samples. Consequently, both the slopes of the regression lines and the *R* ^2^ values abruptly decline, demonstrating that the in‐plane tensile stress almost vanishes in the thin films.

### 2.5 Dielectric Constants of AFE, FE, and PE Thin Films

**Figure 5:**
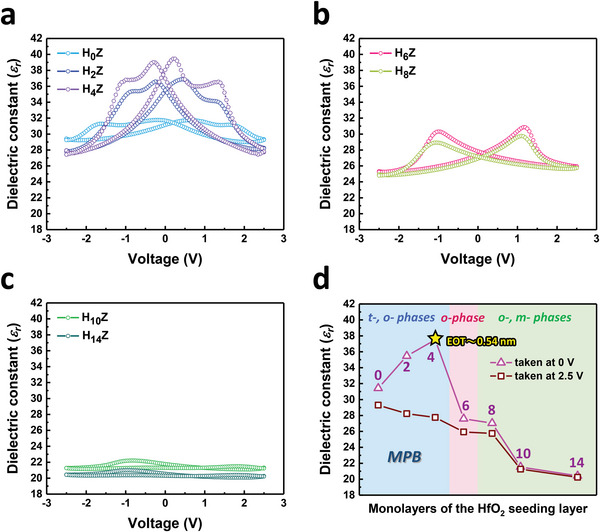
The dielectric property of the HZ thin films. The dielectric constant (*ε_r_*) versus the applied voltage of the a) H_0_Z, H_2_Z, H_4_Z, b) H_6_Z, H_8_Z, and c) H_10_Z, H_14_Z devices. d) The *ε_r_* (taken at 0 and 2.5 V, respectively) versus the monolayers in the HfO_2_ seeding layer of all the samples. High *ε_r_* of ≈31–38 (at 0 V) and ≈28–30 (at 2.5 V) are observed in the H_0_Z, H_2_Z, and H_4_Z samples. The significant difference between *ε_r_* taken at 0 and 2.5 V implicates the presence of MPB due to the coexistence of t‐ and o‐phases. The *ε_r_* degrades to ≈26 (at 2.5 V) as the *T*_HfO2_ increases to 6 and 8 ALD cycles, which is attributed to the dominant o‐phase in the H_6_Z and H_8_Z samples. Further increase in *T*_HfO2_ results in a decrease of *ε_r_* to ≈20–21 (at 2.5 V), which can be explained by the formation of a large amount of the m‐phase in the film.

**Figure** **5**a–c shows the dielectric constant versus voltage (*ε_r_–V*) curves of all the devices, which were extracted from the capacitance versus voltage (*C–V*) measurement. The evolution of the dielectric constant with the number of monolayers in the HfO_2_ seeding layer is plotted in Figure 5d. It has been reported that the *ε_r_* of the t‐, o‐, and m‐phases in ZrO_2_ are ≈46.6, ≈25, and ≈19.7 respectively.^[^ ^29^, ^45^ ^]^ Two pairs of butterfly‐like peaks (the AFE feature) are observed in the *ε_r_–V* characteristics of the H_0_Z, H_2_Z, and H_4_Z samples, and their *ε_r_* (taken at 2.5 V) are in the range of ≈28–30, which suggests the presence of the mixed state of the t‐ and o‐phases. When considering the *ε_r_* at 0 V of the H_4_Z thin film, it is noteworthy that the MPB effect enhances the *ε_r_* up to ≈38 and so a low equivalent oxide thickness (EOT) of only ≈0.54 nm is achieved, which is attractive for high‐k capacitors. With an increase of *T*_HfO2_ over 4 ALD cycles, the *ε_r_–V* curves of the H_6_Z and H_8_Z samples convert to clear butterfly‐like double peaks (the typical FE feature), and their *ε_r_* decreases to about 26 at 2.5 V. The results are highly consistent with the predominant o‐phase in the H_6_Z and H_8_Z layers. The *ε_r_* taken at 2.5 V of the H_10_Z and H_14_Z samples further decreases to ≈20–21, which can be ascribed to the dominant m‐phase in the thin films.^[^ ^2^, ^25^ ^]^ The dielectric properties of all the samples agree well with the electrical and crystalline phase analyses as revealed by the *P–V*, *J–V*, NBD, and XRD measurements.

To gain a deep insight into the origin of MPB which leads to the high *ε_r_* and low EOT of the H_4_Z device, further discussion on the nucleation of the HfO_2_ seeding layer is essential. By examining the *P–V* curves of the H_0_Z, H_2_Z, and H_4_Z devices in Figure 2, the pinched hysteresis loops demonstrate the coexistence of the AFE and FE crystalline phases, that is, the presence of MPB. Notice that the 2*P_r_* of the H_0_Z, H_2_Z, and H_4_Z samples increases with the *T*_HfO2_, suggesting that the HfO_2_ seeding layer may serve as the nucleation sites for the formation of the polar o‐phase in the overlying ZrO_2_ layer. However, the HfO_2_ seeding layer is too thin in the H_2_Z and H_4_Z samples, which results in inhomogeneous nucleation sites and so is insufficient to trigger the overwhelming t‐ to o‐phase transition. This can also be confirmed by Figure 5a,d that only the HfO_2_ seeding layer in the H_2_Z and H_4_Z samples induces the obvious MPB effect,^[^ ^1^, ^46^ ^]^ which leads to a considerable increase in *ε_r_* at 0 V. The dramatic phase transformation to the prominent ferroelectricity in the H_6_Z sample proposes that the 6‐cycle HfO_2_ seeding layer contributes to uniform nucleation sites for polar o‐phase formation.

### 2.6 Correlation between Wake‐Up Effect and Oxygen Vacancies

**Figure 6:**
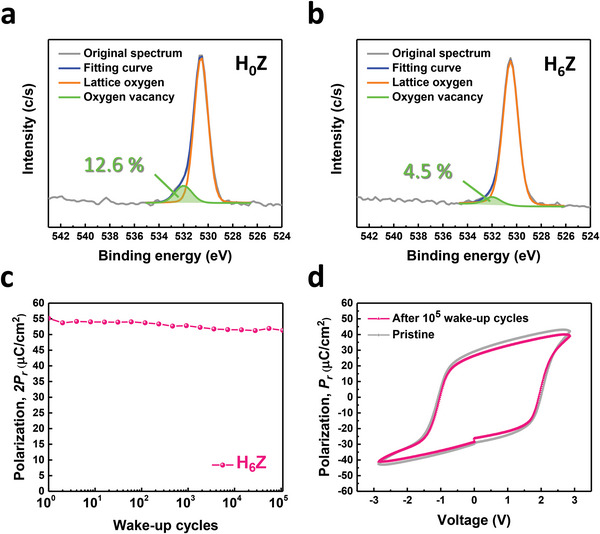
Wake‐up‐free ferroelectricity associated with the suppression of oxygen vacancies. The XPS measurement for the O 1s spectra of the a) H_0_Z and b) H_6_Z samples was conducted to explore the mechanism of the wake‐up‐free behavior. The introduction of the HfO_2_ seeding layer contributes to a decrease in oxygen vacancies from 12.6% to 4.5%. c) The 2*P_r_* versus the wake‐up cycles of the H_6_Z sample. d) The *P–V* curves of the H_6_Z device in the pristine state and the device that has experienced 10^5^ wake‐up cycles. The almost the same 2*P_r_* up to 10^5^ cycles and overlapped *P–V* loops demonstrate the wake‐up‐free property of the H_6_Z sample.

To further explore the effect of the underlying HfO_2_ seeding layer on the ZrO_2_ layer, the XPS measurement was carried out to analyze the O 1s spectra, which can give information on oxygen vacancies in the thin film. **Figure** **6**a,b shows the O 1s XPS spectra of the H_0_Z and H_6_Z samples, respectively. The spectra can be deconvoluted into two main peaks: the one at a lower binding energy of ≈530.5 eV originates from the lattice oxygen, and the other at a higher binding energy of ≈532 eV is correlated with the oxygen vacancies.^[^ ^47^ ^]^ Accordingly, the contents of oxygen vacancies in the films can be estimated by the areas under the fitting curves in the O 1s spectra. One can see that the oxygen vacancies account for ≈12.6% and ≈4.5% of the O 1s spectra in the H_0_Z and H_6_Z samples, respectively, which indicates that the oxygen vacancies are substantially suppressed by inserting the HfO_2_ seeding layer. Since the displacement of atoms tends not to take place in the crystalline region, oxygen vacancies are not prone to present in the area with higher crystallinity.^[^ ^17^ ^]^ Hence the lower amount of oxygen vacancies in the H_6_Z sample can be attributed to the superior crystallinity as revealed by its higher XRD peak intensity than that of the H_0_Z samples. The O 1s XPS spectra of all the samples and the impact of oxygen vacancies on the crystalline phases are discussed in Section S4, Supporting Information.

Figure 6c shows the 2*P_r_* as a function of wake‐up cycles of the H_6_Z device, revealing that the 2*P_r_* nearly keeps constant up to 10^5^ wake‐up cycles. The *P–V* curves of the pristine state and that treated with 10^5^ wake‐up cycles are shown in Figure 6d. The almost identical *P–V* loops indicate the wake‐up‐free characteristic of the H_6_Z sample. It has been widely reported that the local electric field induced by oxygen vacancies will impede the switching of FE dipoles, resulting in weak ferroelectricity at the pristine state.^[^ ^2^, ^16^ ^]^ The redistribution of oxygen vacancies by the cycling electric field during the wake‐up process alleviates the local built‐in electric field in the FE layer, which is the so‐called wake‐up effect for the enhancement of FE properties.^[^ ^16a,d^ ^]^ As shown in the XPS measurement (Figure 6a,b), the amount of oxygen vacancies is drastically restrained by introducing the HfO_2_ seeding layer in the H_6_Z sample. As a result, the excellent FE properties without the need for the wake‐up operation are achieved in the H_6_Z sample. The endurance test of all the FE devices is shown in Section S5, Supporting Information.

### 2.7 The Impact of Grain Size Distribution on MPB‐Correlated Phase Transition

**Figure 7:**
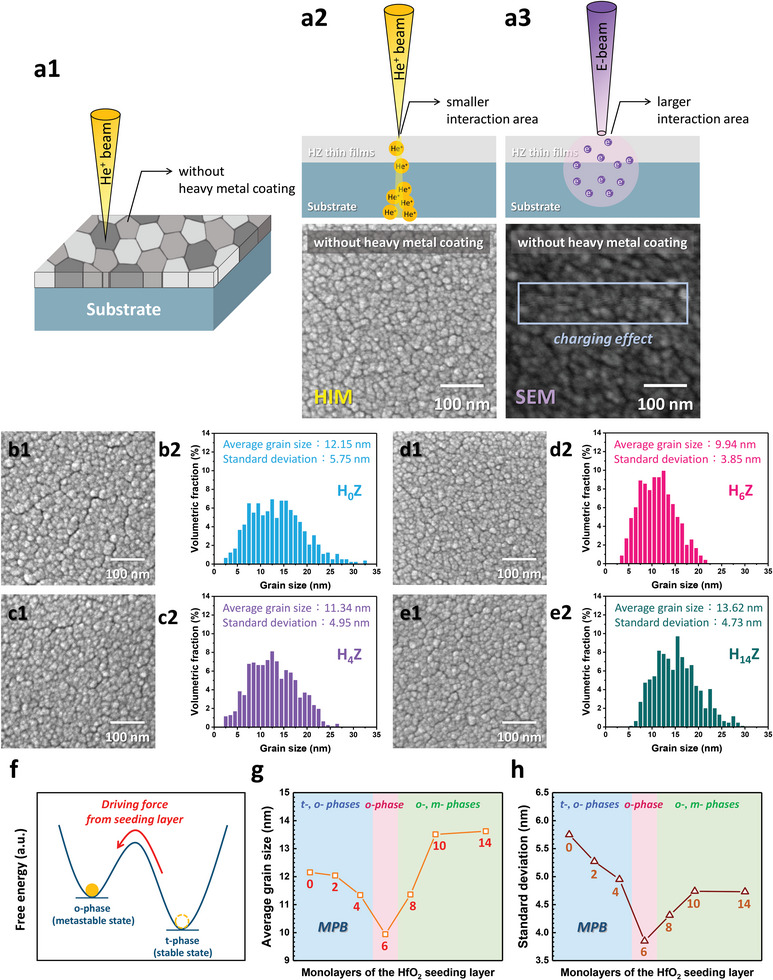
The HIM technology for the extraction of detailed surface grain information of the AFE, FE, and PE thin films. a1–a3) Schematic diagram and the HIM and SEM images of the H_6_Z sample without heavy metal coating. An overwhelmingly clear image of HIM than SEM can be observed. The plane‐view HIM images of the ZrO_2_ surface of the b1) H_0_Z, c1) H_4_Z, d1) H_6_Z, and e1) H_14_Z samples. The distinct grains and grain boundary can be well recognized. (b2), (c2), (d2), and (e2) show the grain size distributions of the H_0_Z, H_4_Z, H_6_Z, and H_14_Z samples, respectively, from which the average and the standard deviation of the grain size can be acquired by the watershed method. f) Schematic of the free energy diagram for stable t‐phase and metastable o‐phase in nanoscale ZrO_2_ thin films, and the driving force is derived from an increase in the surface energy. g) The average and h) the standard deviation of the ZrO_2_ grain size as a function of the monolayers in the HfO_2_ seeding layers. The average and the standard deviation of the grain size are minimal as the number of monolayers in the HfO_2_ seeding layer is 6.

To gain further insight into the roles of the HfO_2_ seeding layer, the HIM was used to characterize the microstructures of the overlying ZrO_2_ layer without any heavy metal coating (**Figure** **7**a1). The surface images of the H_6_Z sample taken by HIM and SEM are shown in Figure 7a2,a3, respectively. A much clear and detail‐rich image can be seen in Figure 7a2, which is attributable to smaller spot size, higher resolution, higher yield of secondary electrons, and alleviated charging effect of HIM than those of SEM. Figure 7b1–e1 reveals the plane‐view HIM images of the H_0_Z, H_4_Z, H_6_Z, and H_14_Z samples, from which high‐contrast and high‐resolution surface images with clear grain boundaries can be observed. Then the watershed method in the Gwyddion software was employed to extract the average grain size and its distribution from ≈700, 750, 870, and 580 grains in the 0.45 µm × 0.45 µm surface area, as shown in Figure 7b2–e2. When the *T*_HfO2_ increases from 0 to 4 and 6 ALD cycles, the average grain size drops from 12.15 to 11.34 and 9.94 nm, and the standard deviation of the grain size decreases from 5.75 to 4.95 to 3.85 nm, respectively, of the H_0_Z, H_4_Z, and H_6_Z samples. As discussed in the previous paragraph, the ≈1 nm (6 ALD cycles) HfO_2_ seeding layer provides the o‐phase for the nucleation of overlying ZrO_2_, which contributes to the smaller average grain size and the more uniform grain size distribution of the ZrO_2_ layer in the H_6_Z sample (Figure 7d2).^[^ ^21b^ ^]^ It has been recognized that the t‐phase predominates in nanoscale ZrO_2_ thin films on account of its lower free energy than that of the o‐phase when the grain size is reduced to below ≈30 nm.^[^ ^2^, ^4^, ^30^, ^48^ ^]^ Since the average grain sizes in the H_0_Z (12.15 nm), H_2_Z (12.04 nm), and H_4_Z (11.34 nm) samples are smaller than 30 nm, the crystallization to the stable t‐phase can be expected as predicted by the thermodynamic theory.^[^ ^4^, ^30^, ^48^, ^49^ ^]^ As shown in Figure 7f, a decrease in the grain size via interposing the HfO_2_ seeding layer leads to an increase in the surface energy, which gives the driving force to overcome the energy barrier for the transformation to metastable o‐phase from the t‐phase in ZrO_2_. This can be appropriately responsible for the correlation between the pronounced ferroelectricity, dominant o‐phase, and smaller average grain size of the H_6_Z sample. On the contrary, the HfO_2_ seeding layer may be too thin to form a continuous nucleation layer in the H_2_Z and H_4_Z samples. The random and non‐uniform nucleation sites bring about a larger variation of the grain size (Figure 7b2,c2) and the coexistence of the t‐ and o‐phases as revealed in the *P–V*, *J–V*, and *ε_r_–V* characteristics (Figures 2 and 5) of the H_0_Z and H_4_Z samples. In contrast, the average grain size of the H_14_Z sample strikingly increases to 13.62 nm as compared with that (9.94 nm) of the H_6_Z sample, which is highly in conformity with the predominance of the m‐phase in the H_14_Z sample (Figure 7e2) as a result of the stabilization of the m‐phase in larger grains.^[^ ^21^, ^30^, ^32^ ^]^ Furthermore, the mixed o‐ and m‐phases in H_14_Z account for the broadening of the grain size distribution with a rise of the standard deviation to 4.73 nm.

Figure 7g,h displays the average and the standard deviation of the grain size versus the monolayers of the HfO_2_ seeding layer, respectively. Both the grain size and its distribution drop to a minimum in the H_6_Z sample. A smaller grain size provides the driving force for the t‐ to o‐phase transition due to an increase in surface energy. On the other hand, the mixture of the t‐ and o‐phases in the H_0_Z, H_2_Z, and H_4_Z samples and the coexistence of the o‐ and m‐phases in the H_8_Z, H_10_Z, and H_14_Z samples, as shown in the *P–V*/*J–V* (Figure 2) and XRD (Figure 3d) measurements, give rise to the increase in the standard deviation of the grain size. The highly pure o‐phase in the H_6_Z sample contributes to the narrower distribution of the grain size. The dependence of the MPB between the t‐, o‐, and m‐phases on the size and distribution of crystalline grains is demonstrated in Figure 7g,h for the first time.

Another noteworthy perspective is the correlation between the grain size and the in‐plane tensile stress. The 6‐cycle HfO_2_ seeding layer results in the minimum grain size of the overlying ZrO_2_ layer as shown in Figure 7g. According to the Volmer–Weber growth of polycrystalline thin films, the intrinsic in‐plane tensile stress (*σ*) can be expressed as follows:^[^ ^44^, ^50^ ^]^

(2) $$\sigma \;=\;\sqrt{\frac{E}{\left(1-\nu \right)}\;\frac{2(2\gamma_{s}-\gamma_{\mathrm{gb})}}{D}}$$

where *E* is Young's modulus, ν is Poisson's ratio, γ_s_ is the surface energy of the film, γ_gb_ is the grain boundary energy, and *D* is the grain size. The equation reveals that a decrease in the grain size gives rise to an increase of the in‐plane tensile stress. Therefore, the minimum grain size in the H_6_Z thin film is also an indispensable factor responsible for significant changes in both the residual stress and the electrical properties of the HZ samples.

## 3 Conclusion

The monolayer engineering on crystalline phases and electrical properties of the nanoscale thin films are well characterized and investigated, including the MPB‐correlated structural change from the t‐ to o‐phases, dielectric properties, defect quantity, and the average size/distribution of crystalline grains. With an increase of 2 monolayers in the HfO_2_ seeding layer, the predominate AFE t‐phase in H_4_Z is converted to the highly pure metastable FE o‐phase in the H_6_Z sample. Thus the excellent ferroelectricity with remarkably high 2*P_r_* ≈ 60 µC cm^−2^ is accomplished in the H_6_Z sample with the FE layer thickness below 6 nm, which is attributed to the introduction of the residual stress and the reduction in the average grain size as evidenced by the XRD and HIM characterizations. Along with the significant FE performance, the wake‐up‐free feature is also achieved in the H_6_Z sample, which can be accounted for by the considerable suppression in the amount of oxygen vacancies due to the increase in crystallinity. The outstanding FE characteristics make the HZ structure very promising and indispensable in next‐generation memory devices. Most importantly, the phase transformation across the MPB can be modulated by the precise control of materials down to the atomic level, which demonstrates the impact of atomic layer engineering and can be expanded to a variety of research and application fields.

## 4 Experimental Section

### Film Deposition and Device Fabrication

As plotted schematically in Figure 1a–c, the Ru/HZ/Ru MIM structures were fabricated on a ≈45 nm TiN adhesion layer on Si substrates. The Ru top and bottom electrodes and the TiN adhesion layer were prepared by magnetron sputtering. The HfO_2_ seeding and ZrO_2_ layers were deposited by plasma‐enhanced ALD. The precursors and the reactant for Hf, Zr, and O were tetrakis‐(dimethylamido)‐hafnium (TDMAHf, Hf[N(CH_3_)_2_]_4_), tetrakis‐(dimethylamido)‐zirconium (TDMAZr, Zr[N(CH_3_)_2_]_4_), and O_2_ plasma, respectively. The deposition temperature of HfO_2_ and ZrO_2_ was 300 °C and the growth rates per cycle of both HfO_2_ and ZrO_2_ were ≈0.16 nm, which were estimated from the cross‐sectional HRTEM images as shown in Figure 3a1,a2. After the deposition of HZ thin films, rapid thermal annealing at 400 °C was conducted in N_2_ ambient for 60 s. Then, the 75 µm × 75 µm square Ru top electrodes were precisely defined on the HZ thin films by the photolithography and lift‐off processes. The layer structure of the HZ sample is shown in the low‐magnification cross‐sectional HRTEM image in Section S6, Supporting Information.

### Electrical Measurements

A semiconductor parameter analyzer (Keithley 4200‐SCS) was employed to characterize the electrical properties including the *P–V*, *J–V*, and *C–V* curves. The *P–V* hysteresis loops and *J–V* curves were obtained from the bipolar triangular voltage waveform at 2 kHz. The *C–V* characteristics were measured with a small AC signal of 30 mV at 1 MHz superimposed on a DC bias sweep. The wake‐up process was performed by the cycling bipolar triangular electric field at 4.7 MV cm^−1^ with a frequency of 2 kHz.

### HRTEM and NBD Characterizations

The cross‐section samples were prepared by the focus ion beam (FEI Helios). Then the HRTEM (FEI Talos F200X) was utilized to take the cross‐sectional images to extract the thickness of HZ layers and the diffraction patterns from the nano electron beam.

### XRD Measurement

Out‐of‐plane, grazing incident, and in‐plane XRD patterns were acquired by the high‐power X‐ray diffractometer with the Cu *K_α_* X‐ray source at *λ* = 0.154 nm (Rigaku TTRAX3) to evaluate the crystalline structures and crystallinity in the nanoscale HZ thin films. The sin^2^ *ψ* method in the grazing incident configuration was conducted by the XRD system (Bruker D8 DISCOVER with GADDS) equipped with the Cu *K_α_* X‐ray source (*λ* = 0.154 nm) at 1.6 kW for the residual stress analysis.

### XPS Analysis

The ratio of oxygen vacancies in the HZ thin films was obtained according to the XPS (PHI VersaProbe scanning microprobe) with monochromatic Al *K_α_* line at 1486.6 eV. The XPS spectra were then deconvoluted using the Gaussian‐Lorentzian peaks by the Multipak software (ULVAC‐PHI, Inc.).

### HIM and SEM Images

The plane‐view images with clear crystalline grains and grain boundaries were taken by helium ion microscopy (Zeiss Orion NanoFab). On the other hand, SEM (JeoL JSM‐7800F Prime) was also used to take the surface image for comparison with the HIM images.

## Conflict of Interest

The authors declare no conflict of interest.

## Author Contributions

M.‐J.C. conceived the idea and coordinated the project. C.‐H.C. and S.‐H.Y. proposed the conceptualization. C.‐H.C. conducted methodology, investigation including electrical properties, XRD analysis, and writing—original draft. C.‐H.C., C.‐Y.C., and J.‐J.S. were responsible for the XPS measurement. C.‐H.C. and T.‐Y.W. contributed to the HIM characterization. M.‐J.C. was in charge of resources, writing—review and editing, supervision, project administration, and funding acquisition. C.‐H.C., Y.‐S.J., and M.‐J.C. were responsible for review questions.

## Supporting information

Supporting Information

## Data Availability

The data that support the findings of this study are available from the corresponding author upon reasonable request.
