# Supplementary material for: Sharp Transformation across Morphotropic Phase Boundary in Sub‐6 nm Wake‐Up‐Free Ferroelectric Films by Atomic Layer Technology
Source: Adv Sci (Weinh). 2023 Sep 27;10(32):2302770. doi: 10.1002/advs.202302770 (PMC10646279; doi:10.1002/advs.202302770)
Supplement: Supplementary file 1 — Supporting Information [file ADVS-10-2302770-s001.pdf]

ADVANCED SCIENCE
Open Access

Supporting Information

for *Adv. Sci.*, DOI 10.1002/advs.202302770

Sharp Transformation across Morphotropic Phase Boundary in Sub-6 nm Wake-Up-Free Ferroelectric Films by Atomic Layer Technology

*Chun-Ho Chuang, Ting-Yun Wang, Chun-Yi Chou, Sheng-Han Yi, Yu-Sen Jiang, Jing-Jong Shyue and Miin-Jang Chen**

Supporting Information

Sharp transformation across morphotropic phase boundary in sub-6 nm wake-up-free ferroelectric films by atomic layer technology

*Chun-Ho Chuang[1], Ting-Yun Wang[1], Chun-Yi Chou[1], Sheng-Han Yi[1], Yu-Sen Jiang[1], Jing-Jong Shyue[2], and Miin-Jang Chen[1]**

[1] Department of Materials Science and Engineering, National Taiwan University, Taipei 10617, Taiwan

[2] Research Center for Applied Sciences, Academia Sinica, Taipei 11529, Taiwan

* Authors to whom any correspondence should be addressed:

E-mail: mjchen@ntu.edu.tw

Section 1: FE characteristics of the H_8Z, $H_{10}Z$, and $H_{14}Z$ devices with higher applied voltages to ensure full switching of polarization

Higher voltages have been applied to measure the *P*-*V*, *J*-*V*, and ε_r-*V* curves of the H_8Z, $H_{10}Z$, and $H_{14}Z$ samples to ensure saturated polarization, as shown in Figure S1. Please be aware that the maximum applied voltage in each curve was chosen to keep an identical maximum electric field of 4.7 MV/cm, and so the maximum voltage drops across the ZrO_2 layers are the same in all the samples because the ZrO_2 layers are equivalent in thickness. It can be seen from Figure S1 that the hysteresis and switching current loops of each sample are highly overlapped with almost the same remanent polarizations and coercive voltages, indicating that the applied voltage of ±2.8 V is sufficient for all the samples to reach fully switched polarization in Figure 2. Figure S1 also shows that the dielectric constants (ε_r) of each sample extracted at the maximum voltages remain nearly identical even if higher voltages were applied in the H_8Z, $H_{10}Z$, and $H_{14}Z$ samples.

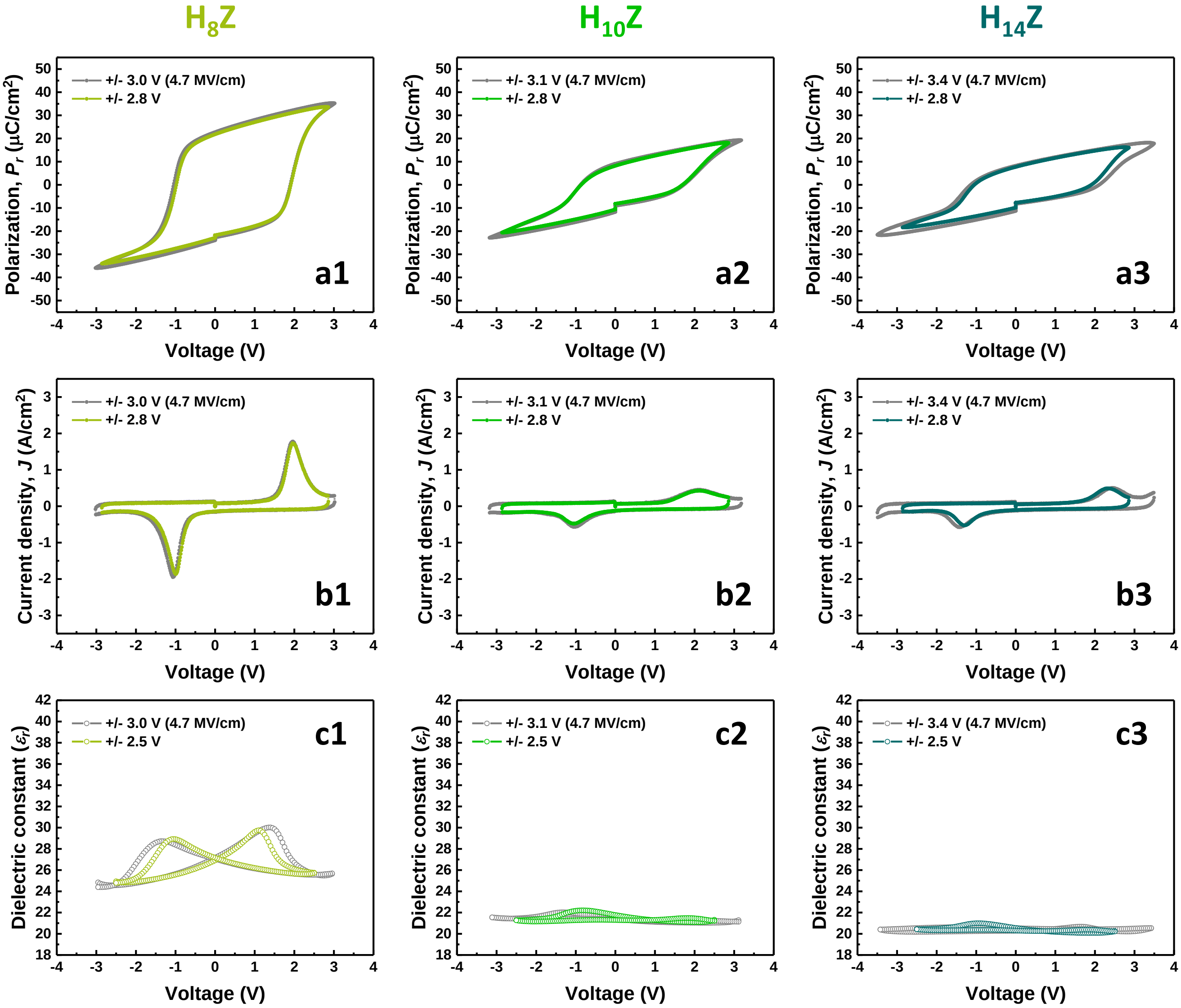

Figure S1 | The pristine-state *P*-*V*, *J*-*V*, and ε_r-*V* curves of the H_8Z, $H_{10}Z$, and $H_{14}Z$ devices. Higher voltages were applied to ensure that the polarization is fully switched. Notice that the maximum applied voltage (3.0 V for H_8Z, 3.1 V for $H_{10}Z$, and 3.4 V for $H_{14}Z$) in each curve was selected to maintain the same maximum electric field of 4.7 MV/cm. As a result, the maximum voltage drops across the ZrO_2 layers are identical in all samples due to the equal thickness of the ZrO_2 layers.

Section 2: The polarity asymmetry of the *P-V* curves

It has been reported that the different work functions of the top and bottom electrodes in MIM devices induce a built-in electric field, which results in the polarity asymmetry of the *P-V* curves [S1]. In this study, the oxygen species during the deposition of the HZ layer by plasma-enhanced ALD and the oxygen diffusion during the post-deposition annealing would result in the formation of the RuO_2 interfacial layer between the Ru bottom electrode and the HZ layer [S2]. As reported in the literature, the work function of RuO_2 (~5.1 eV) is higher than that of the Ru top electrode (~4.6 eV) [S3]. As a result, the difference between the work functions of RuO_2 on the bottom electrode and the Ru top electrode gives rise to the obvious shift of the *P-V* curves toward the positive voltage direction in the H_6Z, H_8Z, $H_{10}Z$, and $H_{14}Z$ samples [S2b].

Section 3: The XRD patterns of the t(101)/o(111) crystallographic planes in the out-of-plane, grazing incident, and in-plane scanning modes

The out-of-plane, grazing incident, and in-plane XRD patterns of all the samples are shown in Figure S2. Upon examination of the out-of-plane, grazing incident, and in-plane XRD patterns in each sample, there are no distinct and regular offsets in the XRD peaks from t(101)/o(111) crystallographic planes except for the H_6Z sample. The lack of noticeable in-plane tensile stress corresponds to the presence of the predominant t-phase in the H_0Z, H_2Z, and H_4Z samples, as well as the predominant m-phase in the $H_{10}Z$ and $H_{14}Z$ samples. As a result, it can be deduced that the presence of significant in-plane tensile stress is favorable for the formation of the FE o-phase in the H_6Z sample.

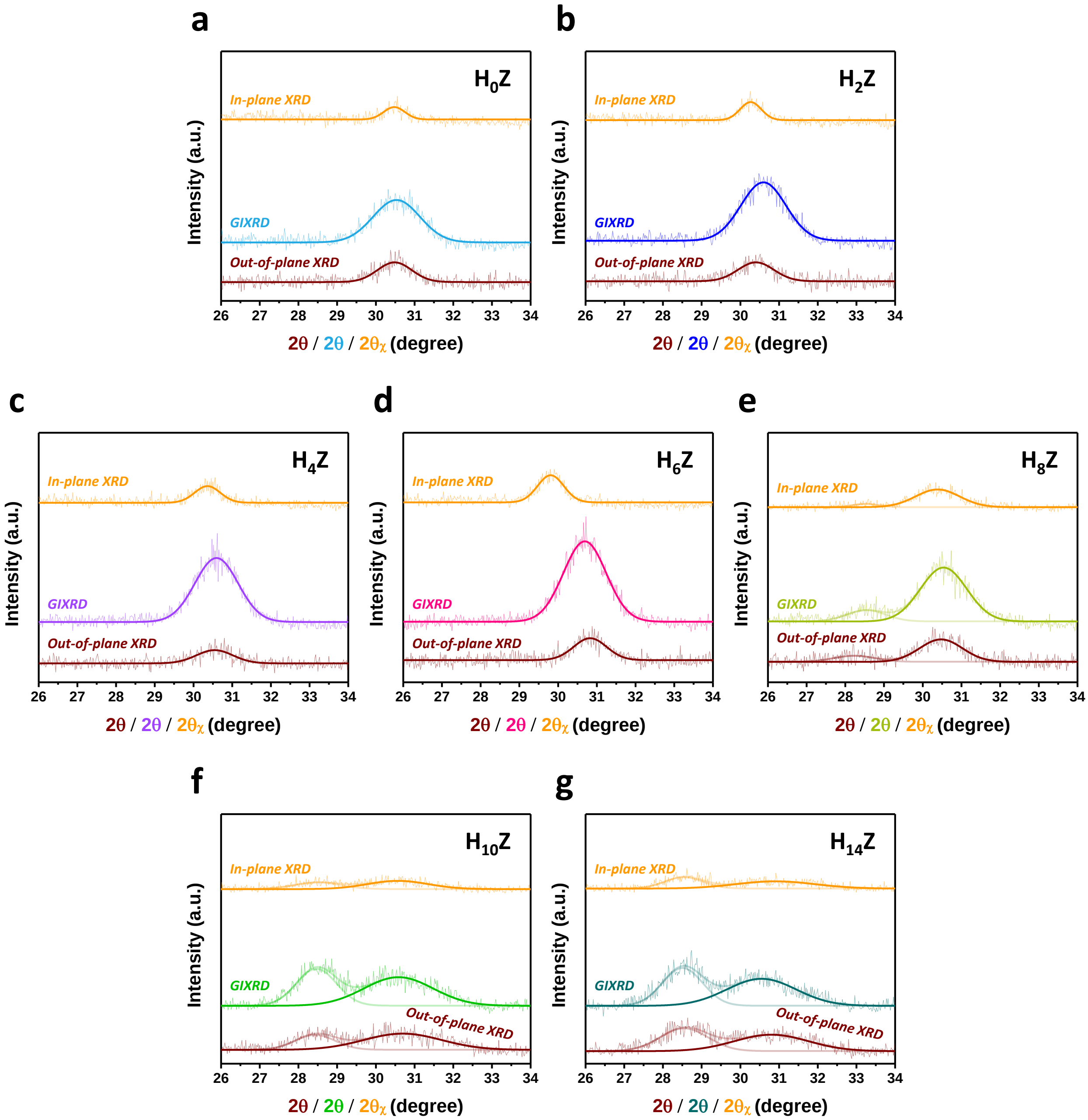

Figure S2 | Out-of-plane, grazing incident, and in-plane XRD patterns of all the samples for the identification of residual stress. The apparent shift from the out-of-plane and grazing incident XRD peaks to the in-plane XRD peak toward the lower diffraction angle can be observed in the H_6Z sample, indicating the presence of significant in-plane tensile stress. Apart from the H_6Z sample, the other samples show minor or irregular offsets among the out-of-plane, grazing incident, and in-plane diffraction peaks.

Section 4: The O 1s XPS spectra of all the samples

Figure S3 shows the O 1s XPS spectra of all the samples. It has been widely reported that the O 1s core level can be classified into three superimposed signals centered at ~530.5 eV, ~532 eV, and ~532.6 eV, which are denoted as O_I, O_{II}, and O_{III}, respectively [S4]. The O_I with the lowest binding energy corresponds to the lattice oxygens (O^{2-}), which are bonded with the adjacent Zr^{4+} or Hf^{4+} in the ZrO_2 or HfO_2 lattices [S5]. On the other hand, the O_{II} signal with a higher binding energy originates from the nearest-neighbor O^{2-} in

the oxygen-deficient region, which is thereby correlated with V_o [S6]. The surface chemisorbed oxygen such as hydroxyl (−OH) and carbonyl (−CO) [S4b, S7] is responsible for the O_{III} with the highest binding energy. However, the O_{III} signal is absent in our case due to the removal of the surface chemisorbed oxygen by the in-situ sputtering before the XPS measurement.

It can be seen from Figure S3 that the incorporation of the HfO_2 seeding layer leads to a significant reduction in oxygen vacancies, and the amount of oxygen vacancies decreases with the HfO_2 thickness. It is worth noting that even just two ALD cycles of HfO_2 can substantially decrease the oxygen vacancies from 12.6% in H_0Z to 5.4% in H_2Z. The HfO_2 seeding layer may suppress the scavenging of oxygen by the bottom electrode, which could be the reason for the decrease in the content of oxygen vacancies in overlying ZrO_2 layer.

Both the first-principle calculation and experimental studies have addressed that the crystalline structures in the HfO_2- and ZrO_2- based thin films can be actually affected by the amount of oxygen vacancies [S8]. Based on the simulation using density functional theory, the formation energies of both the o- and t- phases relative to the m-phase decrease with an increase in the concentration of oxygen vacancies [S8a, S8d]. Furthermore, by tuning the ozone dose during the ALD process of depositing HfO_2- and ZrO_2- based thin films, the effect of oxygen vacancies can be identified experimentally [S8c-e, S9]. Insufficient ozone dose results in a large amount of oxygen vacancies, thereby substantially facilitating the formation of the t-phase [S8c, S8e]. As the ozone dose increases, there exists an optimal window of the quantity of oxygen vacancies for stabilization of the o-phase [S8c, S8e, S8f, S10]. With a further increase in the ozone dose, the m-phase is present since the lack of oxygen vacancies is favorable for the formation of the m-phase [S8c-e]. As shown in Figure S3, the content of oxygen vacancies decreases steadily with an increase in the thickness of the HfO_2 seeding layer, and the corresponding crystalline structure undergoes the transformation from a mixture of t- and o-phases, to predominant o-phase, and ultimately to the coexistence of o- and m- phases, which are highly consistent with the first-principle prediction and experimental results as mentioned above. However, the crystalline phases can be affected not only by oxygen vacancies, but also by other factors such as doping, strain, and surface energy [S10a, S11]. In this study, it has been experimentally demonstrated that the HfO_2 seeding layer has a substantial influence on the phase transition temperature and the strain in the films, both of which also hold considerable significance in the resulting crystalline phase.

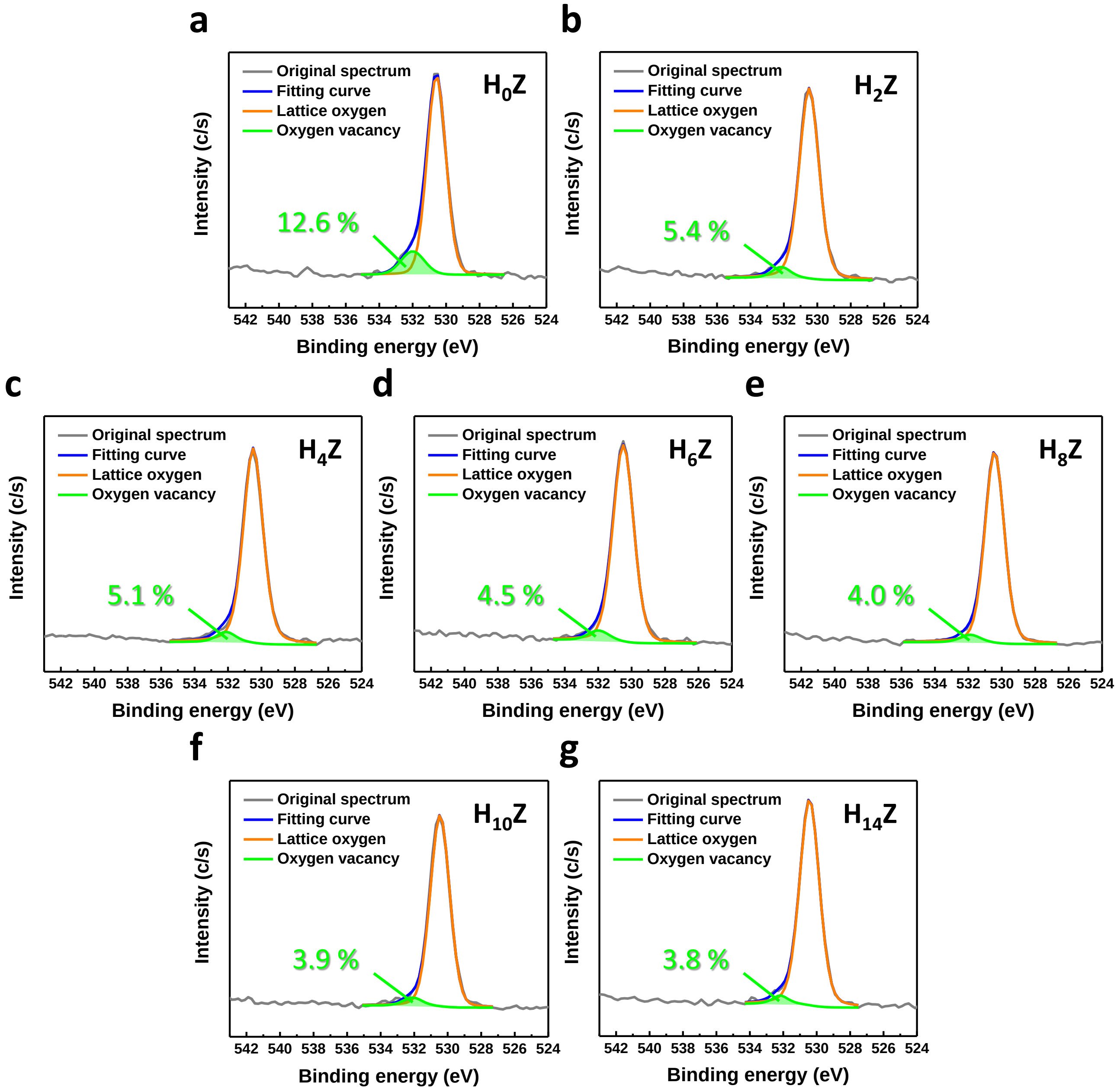

Figure S3 | The O 1s XPS spectra and the contents of oxygen vacancies in all of the samples. The proportions of oxygen vacancies are (a) 12.6% in H_0Z, (b) 5.4% in H_2Z, (c) 5.1% in H_4Z, (d) 4.5% in H_6Z, (e) 4.0% in H_8Z, (f) 3.9% in $H_{10}Z$, and (g) 3.8% in $H_{14}Z$ samples. The HfO_2 seeding layer plays a crucial role in suppressing oxygen vacancies.

Section 5: Endurance and wake-up free performance of all the FE devices

In order to gain a comprehensive understanding of the relevance between the wake-up effect and the quantity of oxygen vacancies, the endurance was examined on all the FE (H_6Z, H_8Z, $H_{10}Z$, and $H_{14}Z$) devices. The endurance test was performed by the cycling bipolar triangular voltage with a magnitude of ±2.8 V at a frequency of 2 kHz. It can be observed from Figure S4 that the $2P_r$ values keep almost identical throughout ~10^5 cycles, indicating the wake-up-free performance of the H_6Z, H_8Z, $H_{10}Z$, and $H_{14}Z$ devices. Because the amount of oxygen vacancies (4.5% to H_6Z, 4.0% to H_8Z, 3.9% to $H_{10}Z$, and 3.8% to $H_{14}Z$) are significantly suppressed by the HfO_2 seeding layer, the wake-up-free feature is highly correlated with the

low contents of oxygen vacancies in the FE layers [S9, S12]. Nevertheless, the $2P_r$ degrades abruptly when the endurance cycles exceed 10^6 for all the devices. Since oxygen vacancies have been proposed to be in favor of the stabilization of the metastable polar o-phase [S8a, S8c, S13], the fatigue in the HZ thin films should be associated with the low amount of oxygen vacancies in the FE thin films.

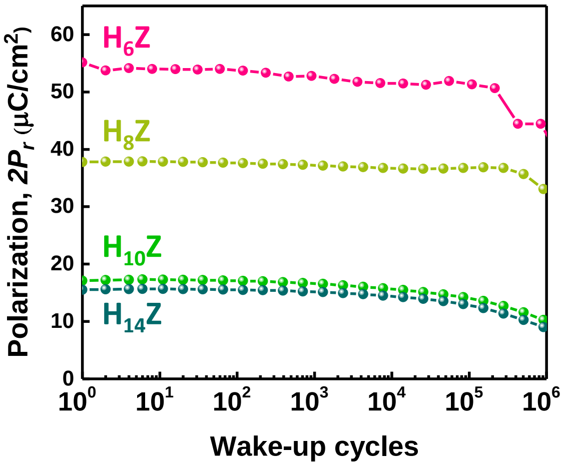

Figure S4 | The endurance and wake-up-free operation of the FE devices. The $2P_r$ values keep nearly constant within 10^5 wake-up cycles, which reveals the wake-up-free ferroelectricity of the H_6Z, H_8Z, $H_{10}Z$, and $H_{14}Z$ devices. After 10^6 cycles, the $2P_r$ begins to degrade in all the samples.

Section 6: The layer structure of the HZ sample

As shown in the cross-sectional HRTEM image in Figure S5, the thicknesses of the Ru top and bottom electrodes are ~46 nm and ~48 nm, respectively. The ~45 nm TiN serves as the adhesion layer to prevent the peeling of the Ru bottom electrode from the Si substrate.

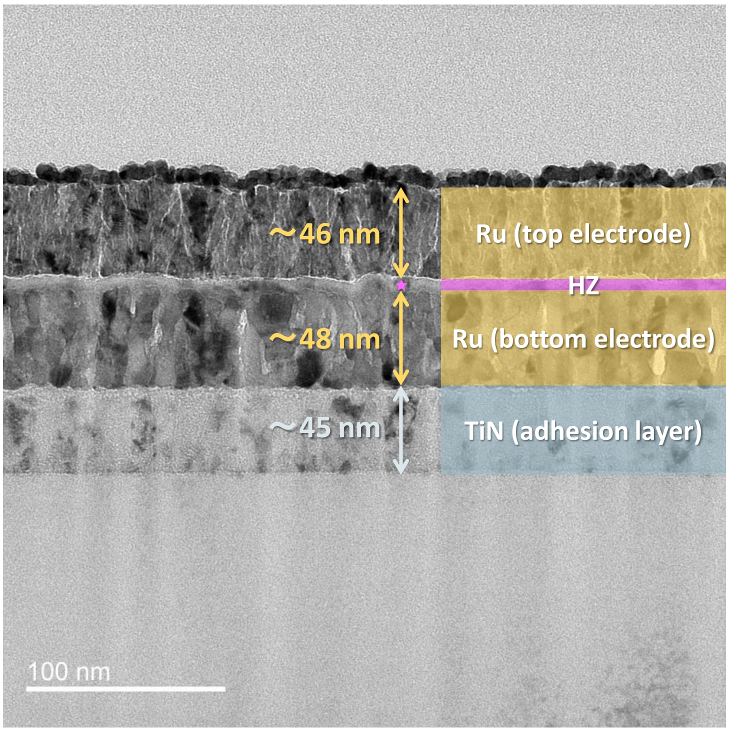

Figure S5 | Low-magnification cross-sectional HRTEM image of the HZ sample.

Reference

[S1] a) R. Cao, Y. Wang, S. Zhao, Y. Yang, X. Zhao, W. Wang, X. Zhang, H. Lv, Q. Liu, M. Liu, *IEEE Electron Device Letters* **2018**, *39* (8), 1207; b) S. S. Fields, S. T. Jaszewski, M. K. Lenox, J. F. Ihlefeld, *Advanced Materials Interfaces* **2023**, *10* (8), 2202232; c) Y. Zhang, Z. Fan, D. Wang, J. Wang, Z. Zou, Y. Li, Q. Li, R. Tao, D. Chen, M. Zeng, *ACS Applied Materials & Interfaces* **2020**, *12* (36), 40510.

[S2] a) G.-J. Choi, S. K. Kim, S.-J. Won, H. J. Kim, C. S. Hwang, *Journal of the Electrochemical Society* **2009**, *156* (9), G138; b) T.-Y. Wang, C.-L. Mo, C.-Y. Chou, C.-H. Chuang, M.-J. Chen, *Acta Materialia* **2023**, *250*, 118848.

[S3] a) E. V. Jelenkovic, K. Tong, *Journal of Vacuum Science & Technology B: Microelectronics and Nanometer Structures Processing, Measurement, and Phenomena* **2004**, *22* (5), 2319; b) K. Fröhlich, K. Husekova, D. Machajdik, J. Hooker, N. Perez, M. Fanciulli, S. Ferrari, C. Wiemer, A. Dimoulas, G. Vellianitis, *Materials Science and Engineering: B* **2004**, *109* (1-3), 117; c) J. Psarouthakis, R. Huntington, *Surface Science* **1967**, *7* (3), 279.

[S4] a) H. W. Cho, P. Pujar, M. Choi, S. Kang, S. Hong, J. Park, S. Baek, Y. Kim, J. Lee, S. Kim, *npj 2D Materials and Applications* **2021**, *5* (1), 46; b) J. H. Kim, Y. J. Jang, J. H. Kim, J.-W. Jang, S. H. Choi, J. S. Lee, *Nanoscale* **2015**, *7* (45), 19144; c) S. Rahimnejad, J. H. He, F. Pan, W. Chen, K. Wu, G. Q. Xu, *Materials Research Express* **2014**, *1* (4), 045044.

[S5] a) Y. Choi, C. Han, J. Shin, S. Moon, J. Min, H. Park, D. Eom, J. Lee, C. Shin, *Sensors* **2022**, *22* (11), 4087; b) T. V. Perevalov, I. P. Prosvirin, E. A. Suprun, F. Mehmood, T. Mikolajick, U. Schroeder, V. A. Gritsenko, *Journal of Science: Advanced Materials and Devices* **2021**, *6* (4), 595.

[S6] a) J. C. Fan, J. B. Goodenough, *Journal of Applied Physics* **1977**, *48* (8), 3524; b) M. Chen, X. Wang, Y. Yu, Z. Pei, X. Bai, C. Sun, R. Huang, L. Wen, *Applied Surface Science* **2000**, *158* (1-2), 134.

[S7] a) J. Felizco, T. Juntunen, M. Uenuma, J. Etula, C. Tossi, Y. Ishikawa, I. Tittonen, Y. Uraoka, *ACS Applied Materials & Interfaces* **2020**, *12* (43), 49210; b) M. M. Can, S. I. Shah, M. F. Doty, C. R. Haughn, T. Fırat, *Journal of Physics D: Applied Physics* **2012**, *45* (19), 195104.

[S8] a) Y. Zhou, Y. Zhang, Q. Yang, J. Jiang, P. Fan, M. Liao, Y. Zhou, *Computational Materials Science* **2019**, *167*, 143; b) M. Hoffmann, U. Schroeder, T. Schenk, T. Shimizu, H. Funakubo, O. Sakata, D. Pohl, M. Drescher, C. Adelmann, R. Materlik, *Journal of Applied Physics* **2015**, *118* (7); c) T. Mittmann, M. Materano, S.-C. Chang, I. Karpov, T. Mikolajick, U. Schroeder, in *2020 IEEE International Electron Devices Meeting (IEDM)* IEEE, **2020**, 18.4. 1-18.4. 4; d) M. Materano, T. Mittmann, P. D. Lomenzo, C. Zhou, J. L. Jones, M. Falkowski, A. Kersch, T. Mikolajick, U. Schroeder, *ACS Applied Electronic Materials* **2020**, *2* (11), 3618; e) B. Xu, L. Collins, K. M. Holsgrove, T. Mikolajick, U. Schroeder, P. D. Lomenzo, *ACS Applied Electronic Materials* **2023**, *5* (4), 2288; f) M. Materano, P. D. Lomenzo, A. Kersch, M. H. Park, T. Mikolajick, U. Schroeder, *Inorganic Chemistry Frontiers* **2021**, *8* (10), 2650.

[S9] A. Kashir, S. Oh, H. Hwang, *Advanced Engineering Materials* **2021**, *23* (1), 2000791.

[S10] a) W. Banerjee, A. Kashir, S. Kamba, *Small* **2022**, *18* (23), 2107575; b) U. Schroeder, M. H. Park, T. Mikolajick, C. S. Hwang, *Nature Reviews Materials* **2022**, *7* (8), 653.

[S11] a) M. H. Park, Y. H. Lee, H. J. Kim, Y. J. Kim, T. Moon, K. D. Kim, J. Mueller, A. Kersch, U. Schroeder, T. Mikolajick, *Advanced Materials* **2015**, *27* (11), 1811; b) Z. Fan, J. Chen, J. Wang, *Journal of Advanced Dielectrics* **2016**, *6* (02), 1630003.

[S12] a) M. Yadav, A. Kashir, S. Oh, R. D. Nikam, H. Kim, H. Jang, H. Hwang, *Nanotechnology* **2021**, *33* (8), 085206; b) T. Onaya, T. Nabatame, M. Inoue, T. Sawada, H. Ota, Y. Morita, *APL Materials* **2022**, *10* (5).
[S13] P. Jiang, Q. Luo, X. Xu, T. Gong, P. Yuan, Y. Wang, Z. Gao, W. Wei, L. Tai, H. Lv, *Advanced Electronic Materials* **2021**, *7* (1), 2000728.
